# Neurological manifestations of acute SARS-CoV-2 infection in a reference hospital in Bahia, Brazil

**DOI:** 10.1016/j.bjid.2025.104542

**Published:** 2025-05-26

**Authors:** Jesângeli de Sousa Dias, Maria Augusta Moreira Rebouças, Lilian Verena da Silva Carvalho, Thais Sampaio Silva, Jair Santana dos Santos, Astrid Xiomara Tatiana Otero Melendez, Carlos Brites

**Affiliations:** a Programa de Pós-Graduação em Medicina e Saúde, Faculdade de Medicina da Bahia, Universidade Federal da Bahia, Salvador, Bahia, Brazil; b Instituto Couto Maia, Salvador, Bahia, Brazil

**Keywords:** COVID-19, Acute encephalopathy, Chemosensory dysfunction, Neutrophil-lymphocyte-ratio, Ethnicity, Outcome

## Abstract

**Background:**

Neurologic manifestations of Coronavirus Disease-19 (COVID-19) have been associated with patients’ disease severity and outcome. This study aimed to describe the frequency and characteristics of the neurological manifestations in a group of hospitalized individuals with COVID-19 and their associations with patient outcomes.

**Methods:**

Patients aged 18 years or older admitted to a local hospital between April and June 2020 with SARS-CoV-2 detected by RT-PCR were included in this retrospective observational study. The characteristics of participants were collected from electronic medical records using a structured questionnaire. A Poisson regression model was used to examine the influence of neurological manifestations on mortality.

**Results:**

A total of 305 participants with COVID-19 were included, with 57.7 % of them presenting neurological symptoms. There were 62 (20.3 %) individuals with acute encephalopathy, with a mean age of 65.5 ± 15.9 years. In this group, higher Prevalence Ratios (PR) of comorbidities (1.6) and severe disease (3.6) were present, predisposing factors for acute encephalopathy. They were also more likely to be admitted to the intensive care unit (3.1) and to die (2.4). The median Neutrophil-Lymphocyte Ratio (NLR) was 7 (Interquartile Range [IQR: 4‒12]). Fifty-two (17 %) participants presented chemosensory dysfunction, with a mean age 53.3 ± 14 years and a lower PR of comorbidity (0.8) than those without. The severe diseases’ PR was slightly higher (1.1), but the PR of ICU admission (0.7), and deaths (0.4) was lower. The LNR was 3.8 (IQR: 2.2–7.8). Poisson regression analysis revealed that severe illness (PR = 3.13), cardiopathy (PR = 1.65), acute encephalopathy (PR = 1.49), diabetes (PR = 1.46), and neutrophil-lymphocyte ratio (PR = 1.04) were associated with death. Conversely, having chemosensory disorders (PR = 0.44) and a prolonged hospital stay (PR = 0.96) were associated with survival.

**Conclusion:**

Patients with acute encephalopathy had more severe forms of COVID-19 and higher mortality. In contrast, chemosensory dysfunction was associated with milder disease manifestations and a better prognosis.

## Introduction

Coronavirus Disease-19 (COVID-19) affected millions of people globally and caused numerous deaths. The initial cases of the disease were identified in Wuhan, China, in December 2019.^1^ and were characterized by severe respiratory disease and high mortality.^2^^,^^3^ As the number of cases increased, more disease characteristics became evident. Mao et al. were the first to report nervous system manifestations associated with COVID-19.^4^ Following them, several studies worldwide have been published, revealing varying frequencies and severities of neurological findings.^5^, ^6^, ^7^, ^8^ Some studies indicate an association between neurological manifestations and outcomes.^9^, ^10^, ^11^

Brazil was severely impacted by the pandemic, with millions of infected individuals and countless deaths. A local report associated a higher risk of being infected with characteristics related to social vulnerability,^12^ and certain regions, particularly in the north, experienced higher incidence and mortality rates.^13^ The risk of death was greater among Black individuals, those with a multiracial background, and indigenous people.^14^ How these aspects could influence neurologic manifestation is unclear.

We studied hospitalized patients infected with SARS-CoV-2 to investigate the frequency and characteristics of neurological involvement by COVID-19 in the population of a local hospital during the beginning of the first pandemic wave, as well as the potential impact of neurologic involvement on patient outcomes.

## Methods

### Study design, participant selection, and settings

This retrospective observational study enrolled all patients infected with SARS-CoV-2 who were hospitalized in a public hospital from April 1 to June 1, 2020. This center is a reference for the treatment of patients with infectious diseases in Bahia, Brazil. During the pandemic, it was dedicated to caring for patients with COVID-19. Most of the admitted patients came from other health services in Bahia. We included participants aged 18-years or older in the study with a PCR-confirmed SARS-CoV-2 infection. The samples were collected from either the nasopharynx or oropharynx. We did not include participants who died, were discharged or transferred to other hospitals within the first 24 hours after admission, who did not undergo the RT-PCR test or had a negative test result. Participants with incomplete medical records and those diagnosed with post-COVID-19 on admission were excluded.

The Institutional Ethics and Research Committee approved this study (protocol number 32856120.7.0000.0046), and patient consent was waived since there was no direct contact between participants and researchers.

### Data collection and definitions of variable

A structured questionnaire was used to gather participant information from electronic medical records. Data collection included demographic characteristics, comorbidities, COVID-19 symptoms, neurological symptoms or signs observed both before and during hospitalization (headache, dizziness, loss of smell, loss of taste, myalgia, seizure, motor deficit, gait impairment, bladder and bowel dysfunction, sensory deficit, cranial nerve deficit, and change in mental status such as confusion, disorientation, agitation, reduced consciousness level), the need for Intensive Care Unit (ICU) admission, any support interventions required, the Length of Stay (LOS) in the ICU, the overall in-hospital LOS, and occurrences of deaths. Additionally, the results of laboratory tests (hemogram, c-reactive protein, lactate dehydrogenase, blood urea nitrogen, creatinine, electrolytes, glucose, calcium, liver function test, prothrombin time, activated partial thromboplastin clotting time), conducted upon admission, as well as any Cerebrospinal Fluid (CSF) studies and cranial imaging, including Computed Tomography (CT) or Magnetic Resonance Imaging (MRI) were documented.

Participants were classified into two categories based on their level of education: those with 0 to 9 years of schooling and those with 10 or more years. Race and ethnicity were categorized according to five groups defined by the Brazilian Institute of Geography and Statistics (IBGE): *Branco* (White), *Preto* (Black), *Amarelo* (East Asian), *Indígena* (Indigenous), and *Pardo* (mixed ethnicity).^15^

Disease severity was defined according to the WHO criteria from 2020.^16^ Individuals classified as asymptomatic, mild, or moderate were grouped as having no severe disease, while those with more serious conditions were categorized as having severe disease.

Neurological signs and symptoms were organized into diagnostic categories whenever possible. To classify conditions such as myelopathy, polyneuropathy, and myopathy, we reviewed neurological examinations documented in medical records. We utilized previously established criteria to define stroke,^17^ encephalitis,^18^ and acute encephalopathy.^19^ The group with chemosensory dysfunction was defined including patients who reported impairment in smell or taste.

We used the Neutrophil-Lymphocyte Ratio (NLR) as the laboratory index of the severity of inflammation.^20^ The outcomes were the frequency of death within 30 days of hospital admission.

### Statistical analysis

Categorical data was reported in absolute and relative frequency, while continuous values were expressed as means with Standard deviation (ST) or median with Interquartile Range (IQR). To compare differences for independent groups between categorical variables, either the Chi-Square (χ^2^) test or Fisher's exact test was applied as appropriate. In addition, the Student *t*-test or the Mann-Whitney *U* test was used to analyze differences for independent groups between continuous values, depending on their distribution. A p-value ≤0.05 was considered statistically significant.

The Prevalence Ratio with a 95 % Confidence Interval (PR, 95 % CI) was calculated to estimate the association between independent variables and main neurological categories. A Poisson regression analysis examined potential confounding factors that could interfere with the association between the main neurological categories and death. Variables related to death with *p* ≤ 0.2 in bivariate analysis were inserted in the saturated model. Following that, variables with a *p* ≤ 0.05 in the saturated model were inserted in the adjusted model. The Omnibus test was used to evaluate the model adjustment. The model is considered reliable when the p-value is < 0.001, along with a reduction in the Akaike Information Criterion (AIC) value. Statistical analysis was performed using the IBM SPSS statistical package (v.25.0) and OpenEpi software (OpenEpi: Open-Source Epidemiologic Statistics for Public Health, Version. www.OpenEpi.com, 2013/04/06).

## Results

**Fig. 1:**
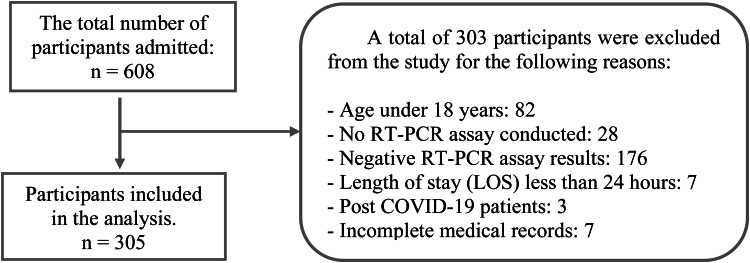
**Flowchart of sample selection.** Description: This flowchart details the reasons for excluding some data points from the sample analyzed in the study.

During the period, 608 patients with confirmed or suspected SARS-CoV-2 infection were hospitalized. For this study, the data records of a sample including 305 of those patients were considered. Fig. 1 presents a flowchart detailing the sample selection.

In this sample, 209 participants were classified as having severe disease at the time of admission. Additionally, 15 participants were asymptomatic and hospitalized for other reasons, 40 had mild disease, and 41 had moderate disease. The frequency of comorbidities was high. The most common symptoms of COVID-19 were cough, dyspnea, and fever, whereas nasal obstruction and sneezing occurred in less than 2 % of cases. The frequency of neurological symptoms or signs observed is summarized in Table 1.

**Table 1:** Frequency of neurological symptoms/signs in patients with COVID-19.

	Total	Upon admission	During disease
**Any symptom/sign**	176 (57.7)	142 (46.6)	34 (11.1)
**Altered mental status**	62 (20.3)	29 (9.5)	33 (10.8)
**Myalgia**	60 (19.6)	59 (19.3)	1 (0.3)
**Headache**	51(16.7)	40 (13.1)	11 (3.6)
**Smell loss**	43 (14.1)	42 (13.8)	1 (0.3)
**Taste loss**	28 (9.2)	27 (8.9)	1 (0.3)
**Dizziness**	8 (2.6)	3(1)	5 (1.6)
**Seizure**	8 (2.6)	2 (0.7)	6 (2)
**Deficit motor in all limbs**	12 (3.9)	2 (0.7)	10 (3.3)
**Focal motor deficit**	3(1)	2 (0.7)	1 (0.3)

We identified one participant diagnosed with encephalitis; however, no SARS-CoV-2 was detected in the Cerebrospinal Fluid (CSF) through RT-PCR testing. In another participant who would later die, a CT scan revealed small areas of intracerebral hemorrhage in the left parietal lobe, with undetermined cause. Two other participants were diagnosed with ischemic stroke. One of them was an 83-year-old man with a medical history of diabetes and hypertension, who was admitted after 8-days of respiratory symptoms and 24 hours with a reduced level of consciousness. A cranial CT showed a right pontine hypodensity area. No further investigations were carried out. This participant died four days after being admitted. The other one was a 47-year-old woman with an unremarkable medical history, admitted with 8-days of respiratory symptoms followed by headache, acute onset of left arm and leg weakness, and numbness followed by a decreased level of consciousness. A cranial CT showed a large area of hypodensity with loss of gray-white matter differentiation involving the right frontal, temporal, and parietal lobes, basal nucleus, and internal capsule. CT angiography of cervical and cranial vessels showed a thrombus occluding her right middle cerebral artery, originating from the right carotid artery. No atherosclerotic sign was detected in these vessels. Transthoracic echocardiography was normal. No additional investigation was carried out during hospitalization. She was discharged.

Acute encephalopathy was identified in 20.3 % (62/305) of cases (for a detailed description, see Table 2). This manifestation was presented upon admission in 46.8 % (29/62) of participants, with the remaining cases developing it later. Among those admitted with symptoms of acute encephalopathy, 65.5 % (19/29) of them required supplemental oxygen on admission, and the time elapsed from COVID-19 symptom onset to admission was 5.5 (IQR: 3.25–8) days.

**Table 2:** Baseline characteristics of patients with COVID-19 and acute encephalopathy.

	Total (*n* = 305)	Acute Encephalopathy		PR (95 % CI)	p
		Yes (*n* = 62)	No (*n* = 243)		
**Demographics data**					
Age years, mean (ST)	57.7 (16.1)	65.5 (15.9)	55.7 (15.5)		<0.001
Male sex, n (%)	170 (55.7)	32 (18.8)	138 (81.2)	0.9 (0.5‒1.3)	
Race, (%)^a^					
Black	51 (16)	9 (16.6)	42 (82.4)	0.9 (0.4‒1.6)	
*Pardos*	226 (74.1)	45 (19.9)	181 (80.1)	1 (0.6–1.7)	
White	18 (5.9)	5 (27.8)	13 (72.2)	1.4 (0.6–3.1)	
9-years or less of schooling, n (%)^b^	143 (46.9)	33 (23.1)	110 (76.9)	1.4 (0.9‒2.4)	
**Clinical presentation, n (%)**					
Fever	194 (63.6)	43 (22.2)	151 (77.8)	1.3 (0.8‒2.1)	
Cough	205 (67.2)	33 (16.1)	172 (83.9)	0.5 (0.3‒0.9)	<0.001
Dyspnea	201 (65.9)	38 (18.9)	163 (81.1)	0.8 (0.5‒1.3)	
Severe COVID-19	209 (68.5)	55 (26.3)	154 (73.7)	3.6 (1.7‒7.6)	<0.001
**Comorbidity, n (%)**					
Any comorbidities	254 (83.3)	55 (21.7)	199 (78.3)	1.6 (0.8‒3.3)	
Hypertension	161 (52.8)	39 (24.2)	122 (75.8)	1.5 (0.9‒2.4)	
Diabetes	97 (31.8)	26 (26.8)	71 (73.3)	1.5 (1‒2.4)	0.05
Obesidade	69 (22.6)	13 (18.8)	56 (81.2)	0.9 (0.5‒1.6)	
Prior stroke	23 (7.5)	10 (43.5)	13 (56.5)	2.3 (1.4‒4)	0.04
Cardiopathy	42 (13.8)	13 (31)	29 (69)	1.7 (1‒2.8)	
Pneumopathy	20 (6.6)	3 (15)	17 (85)	0.7 (0.2‒2.1)	
Dementia	15 (4.9)	7 (46.7)	8 (53.3)	2.5 (1.7‒4.5)	0.001
Chronic renal failure	32 (10.5)	7 (21.9)	25 (78.1)	1.1 (0.5‒2.2)	
ICU **admission, n (%)**	173 (56.7)	50 (28.9)	123 (71.1)	3.1 (1.8‒5.7)	<0.001
**LOS ICU days, median (IQR)**	8.5 (5‒14)	12.5 (6‒23.2)	8 (4.7‒12)		0.002
**LOS in-hospital days, median, (IQR)**	10 (6‒16)	13 (8.7‒25.2)	9 (6‒15)		<0.001
**Deaths, n (%)**	108 (35.4)	35 (32.4)	73 (67.6)	2.4 (1.5–3.7)	<0.001

a Information about ethnicity was available for 297 medical records. There were 3 missing values in the acute encephalopathy group. There were 5 missing value and 2 (0.7 %) Asian participants in the other group.

b Information about education level was available to 291 participants. There were 21 participants with no formal education, 122 with 1 to 9-years of schooling, 127 with 10 to 12-years of schooling, and 21 up 12-years. Data were missing for 6 participants in the acute encephalopathy group and 8 participants in the other group. IQR, Interquartile Range; LOS ICU, Length of Stay in Intensive Care Unit; LOS in-hospital, Length of Stay in hospital; PR, Prevalence Ratio; 95 % CI, 95 % Confidence Interval.

The group with acute encephalopathy had a higher NLR upon admission (7, IQR: 4–12 vs*.* 5.3, IQR: 2.7–8.4, *p* < 0.002) than those without acute encephalopathy. A Cranial CT was obtained in 17.7 % (11/62) of these participants. The result was normal in 4 four cases; 1 case showed hydrocephalus, and 1 showed right occipital gliosis. The remainder showed brain atrophy, microangiopathy, an unspecific hypodensity area, and intracranial vessel calcification.

During hospitalization, this group required more invasive mechanical ventilation (PR = 3.21, 95 % CI: 2.11–4.88, *p* = 0.001), renal replacement therapy (PR = 2.56, 95 % CI: 1.65–3.98, *p* = 0.01), and vasoactive amine interventions (PR = 4.06, 95 % CI: 2.52–6.54, *p* = 0.001), as well as higher use of antibiotic therapy (PR = 2.49, 95 % CI: 1.55–4.01, *p* = 0.001).

The group with chemosensory dysfunction had a lower NLR (3.8, IQR: 2.2–7.8 vs. 6.1, IQR: 3–9.1, *p* = 0.04) than those without. During ICU stay, their requirement was lower for invasive mechanical ventilation (PR = 0.7, 95 % CI: 0.4–1.4, *p* = 0.32), renal replacement therapy (PR = 0.70, 95 % CI: 0.39–1.26, *p* = 0.26), vasoactive amine support (PR = 0.64, 95 % CI: 0.36–1.15, *p* = 0.15), and antibiotic needs (PR = 0.56, 95 % CI: 0.33–0.98, *p* = 0.03). For a detailed description of this group, see Table 3.

**Table 3:** Baseline characteristics of patients with COVID-19 and chemosensory dysfunction.

	Total (*n* = 305)	Chemosensory dysfunction		PR (95 % CI)	p
		Yes (*n* = 52)	No (*n* = 253)		
**Demographics data**					
Age years, mean (ST)	57.7 (16.1)	53.3 (14)	58.6 (16.3)		0.03
Male sex, n (%)	170 (55.7)	24 (14.1)	146 (85.9)	0.7 (0.4‒1.2)	
Race, n (%)^a^					
Black	51 (16.7)	8 (15.7)	43 (84.3)	0.9 (0.4‒1.8)	
*Pardos*	226 (74.1)	39 (17.3)	187 (82.7)	1 (0.6‒1.8)	
White	18 (5.9)	3 (16.7)	15 (83.3)	1 (0.3‒2.8)	
9-years or less of education, n (%)^b^	291 (95.4)	18 (35.3)	125 (52.1)	0.6 (0.3‒0.9)	
**Clinical presentation, n (%)**					
Fever	194 (63.6)	39 (20.1)	155 (79.9)	1.7 (0.9‒3.1)	
Cough	205 (67.2)	40 (19.5)	165 (80.5)	1.6 (0.9‒3)	
Dyspnea	201 (65.9)	37 (18.4)	164 (81.6)	1.3 (0.7‒2.2)	
Severe COVID-19	209 (68.5)	37 (17.7)	172 (82.3)	1.1 (0.6‒2)	
**Comorbidity, n (%)**					
Any comorbidity	254 (83.3)	42 (16.5)	212 (83.5)	0.8 (0.4‒1.6)	
Hypertension	161 (52.8)	23 (14.3)	138 (85.7)	0.7 (0.4‒1.2)	
*Diabetes*	97 (31.8)	13 (13.4)	84 (86.6)	0.7 (0.4‒1.3)	
*Obesidade*	69 (22.6)	13 (18.8)	56 (81.2)	1.1 (0.6‒2)	
Prior stroke	23 (7.5)	0	23 (100)	‒	0.019
Cardiopathy	42 (13.8)	7 (16.7)	35 (86.3)	1 (0.5‒2)	
Pneumopathy	20 (6.6)	2 (10)	18 (90)	0.6 (0.1‒2.2)	
*Dementia*	15 (4.9)	1 (6.7)	14 (93.3)	0.4 (0.7‒2.6)	
Chronic renal failure	32 (10.5)	3 (9.4)	29 (90.6)	0.5 (0.2‒1.6)	
ICU **admission, n (%)**	173 (56.7)	25 (14.5)	148 (85.5)	0.7 (0.4‒1.1)	
**LOS ICU days, median (IQR)**	8.5 (5‒14)	12 (6.5‒16)	8 (5‒13)		0.04
**LOS in-hospital days, median (IQR)**	10 (6‒16)	11 (7‒16)	10 (6‒16)		
**Death, n (%)**	108 (35.4)	9 (8.3)	99 (91.7)	0.4 (0.2‒0.7)	0.003

a Information about ethnicity was available for 297 medical records. There were 1 an Asian participant in each group. There were 1 missing value in the chemosensory dysfunction group, and 7 in the other group.

b Information available to 291 medical records. There were 21 participants with no formal education, 122 with 1 to 9 years of schooling, 127 with 10 to 12 years of schooling, and 21 up 12 years. Data were missing for 1 participant in the chemosensory dysfunction group and 13 participants in the other group. IQR, Interquartile Range; LOS ICU, Length of stay in Intensive Care Unit; LOS in-hospital, Length of Stay in hospital); PR, Prevalence Ratio; 95 % CI, 95 % Confidence Interval.

A total of 108 (35.4 %) participants died, the majority (102) within 30 days. In the group with acute encephalopathy, 34 (97.1 %) deaths occurred within 30 days, while 7 (77.8 %) were recorded in the group with chemosensory dysfunction. There was no statistically significant difference in the frequency of racial and educational background between those who died and those who did not.

A Poisson regression analysis identified several variables significantly associated with death within 30 days of admission: severe disease, heart disease, acute encephalopathy, diabetes, and NLR value. No association was found with hypertension. Moreover, having chemosensory disorders and a long stay in hospital were associated with survival (see Table 4). The results of the Omnibus test assessed the model's goodness of fit, which produced a p-value of less than 0.001, along with a reduction in the Akaike Information Criterion (AIC) from 384.264 to 380.956.

**Table 4:** Poisson regression analysis of variables associated with death in 30 days.

	Saturated model			Adjusted model		
	PR	95 % CI	p	PR	95 % CI	p
**Age**	1.01	0.99‒1.02	0.32			
**Hypertension**	0.98	0.67‒1.42	0.92			
**Diabetes**	1.44	1.04‒1.99	0.03	1.46	1.09‒1.95	0.011
**Cardiopathy**	1.59	1.11‒2.29	0.01	1.65	1.62‒2.36	0.005
**NLR**	1.04	1.02‒1.06	<0.001	1.04	1.02‒1.06	<0.001
**COVID-19 severity**	3.18	1.80‒5.61	<0.001	3.13	1.77‒5.54	<0.001
**LOS**	0.97	0.95‒0.98	<0.001	0.96	0.95‒0.98	<0.001
**Acute encephalopathy**	1.41	1.01‒1.97	0.04	1.49	1.10‒2.03	0.011
**Chemosensory dysfunction**	0.08	0.04‒0.18	0.02	0.44	0.23‒0.84	0.013

PR, Prevalence Ratio; 95 % CI, 95 % Confidence Interval; NLR, Neutrophil-Lymphocyte Ratio; LOS, Length of Stay in hospital.

Of note, regarding neurological investigation, 21 (6.9 %) participants underwent cranial CT scans, 3 (1 %) had cranial CT angiography, and 4 (1.3 %) had lumbar punctures with Cerebrospinal Fluid (CSF) analysis. Among the cases of acute encephalopathy, a cranial CT was obtained in 17.7 % (11/62) of these participants. The result was normal in four cases; one case showed hydrocephalus, and one showed right occipital gliosis. The remainder showed brain atrophy, microangiopathy, an unspecific hypodensity area, and intracranial vessel calcification. Two participants carried out lumbar puncture with CSF analysis with a normal result. Furthermore, there were missing values in most laboratory analyses upon admission. For a description of laboratory analysis results, see Table 5. A complete blood count was obtained for 304 participants, allowing the calculation of the neutrophil-lymphocyte ratio.

**Table 5:** Result of laboratory investigation from 305 individuals with SARS-CoV-2 infection.

	Valid results a	Total b	Acute encephalopathy		p	Chemosensory dysfunction		p
			Yes	No		Yes	No	
**Hemoglobin, g/L**	304 (99.7)	12.7 (10.9‒13.8)	12.6 (9.3‒13.6)	12.7 (11.1‒13.9)		13 (11.8‒13.9)	12.6 (10.8‒13.8)	
**Hematocrit, %**	304 (99.7)	37 (32‒40)	37 (27.8‒39.8)	36.9 (32.3‒40.2)		38.1 (34.7‒39.9)	36.6 (31.1‒40.2)	
**White blood cell, /μL**	304 (99.7)	8.87 (6.32‒11.87)	10.78 (7.89–13.41)	8.30 (6.17‒11.30)	0.001	8.02 (5.98‒10.96)	9.02 (6.66–12.10)	
**Neutrophil Count, /μL**	304 (99.7)	6.75 (4.17–9.46)	8.41 (6.04–10.99)	6.15 (4.01–8.87)	0.001	5.59 (3.64–8.82)	6.83 (4.31–9.63)	
**Lymphocyte count, /μL**	304 (99.7)	1.27 (0.88–1.78)	1.11 (0.82–1.53)	1.31 (0.91–1.90)		1.22 (0.98–2.02)	1.28 (0.84–1.76)	
**Platelet count, 10^3^/μL**	304 (99.7)	253.00 (189.25‒344.75)	264.00 (194.00–344.00)	246.50 (184.75‒345.25)		235.50 (198.25–343.50)	256.00 (187.25–345.75)	
**CGB, mg/dL**	276 (90.5)	127 (96.7–174)	127 (96.7–174)	109 (90.7–146.5)	0.02	107 (92–190)	110 (90–145)	
**Blood urea nitrogen, mg/dL**	304 (99.7)	36 (26‒72)	50 (31.7‒83.2)	33.5 (26‒65.2)	0.002	28(21‒35.5)	39.5 (27‒77)	0.000
**Creatinine, mg/dL**	305 (100)	0.8 (0.7‒1.2)	0.9 (0.7‒1.2)	0.8 (0.7‒1.3)		0.7 (0.6–1)	0.9 (0.7–1.3)	0.023
**Sodium, mEq/**	282 (92.4)	139 (136‒142)	139 (136‒142)	139 (136‒142)		139 (135.7–141)	139 (136–142)	
**Potassium mEq/L**	281 (92.1)	4.4 (4‒5)	4.5 (4.2‒5.1)	4.4 (4‒5)		4.3 (4–4.9)	4.5 (4.1–5)	
**Magnesium, mg/dL**	78 (25.6)	2.1 (2–2.3)	2.1 (2–2.3)	2 (2–2.3)		2 (2–2.5)	2.1 (2–2.3)	
**Calcium, mg/dL**	96 (31.5)	8.8 (8.4–9.4)	8.6 (8.3–9.2)	8.6 (8.3–9.2)		9.2 (9–9.4)	8.7 (8.3–9.4)	
**Alanine aminotransferase, U/L**	265 (86.9)	45 (31‒78)	48 (34‒74)	43,5 (30.7‒79)		53 (31.5–82.5)	44 (31–76.5)	
**Aspartate aminotransferase, U/L**	260 (85.2)	40 (24.2‒65.5)	35 (22.5‒53.5)	43 (25‒68)		44 (28–87.5)	40 (23.2–62)	
**Alkaline phosphatase, U/L**	88 (28.8)	239.5 (185.5–338.5)	274 (201–390)	231 (182–325)		212 (181.2–362.2)	244.5 (187–338.5)	
**Gamma-glutamyl transferase, U/L**	87 (28.5)	87 (59–223)	102 (54.5–239.5)	112.5 (58–202)		91 (44–136)	121.5 (60.2–241)	
**Albumin, g/L**	163 (53.4)	3.3(3‒3.6)	3.3(3‒3.5)	3.3(3‒3.7)		3.4 (3.2–3.9)	3.3 (3‒3.5)	0.029
**C-reactive protein, mg/dL**	206 (67.5)	10.6 (5.7‒17.3)	11.7 (7.9–20.6)	9.9 (4.6–16.4)	0.025	11.5 (2.8–18.1)	10.3 (5.6‒17.3)	
**Lactate dehydrogenase, U/L**	103 (33.8)	717 (567–929)	762 (602–1136)	681 (558–912)		798 (501–912)	708 (581–944)	
**Prothrombin time, %**	247 (81)	85 (71‒96)	82 (70.7‒92.5)	85 (71.5‒98)		88 (77.2–96.7)	83 (71–96.5)	
**International normalized ratio**	246 (80.6)	1.1 (1‒1.2)	1.1 (1‒1.2)	1.1 (1‒1.2)		1.1 (1–1.1)	1.1 (1–1.2)	
**APTT, seconds**	232 (76.1)	31.2 (28.3–34)	31 (27.3–33.8)	31.2 (28.5–34.2)		31.2 (27.7–33.1)	31.2 (29–34.2)	

Note: the results are presented as median with interquartile range or absolute and relative frequency.

a There are variable missing values among those laboratory investigation. This column presents the number of laboratory investigation carried out on admission to the hospital.

b This column presents all results obtained. APTT, Activated Partial Thromboplastin clotting Time; CBG, Capillary Blood Glucose.

## Discussion

This retrospective observational study revealed that 46.6 % of patients with SARS-CoV-2 who were hospitalized displayed at least one neurological symptom upon admission. The most common symptoms included myalgia, loss of smell, headache, changes in mental status, and loss of taste, consistent with previous reports.^21^ Acute encephalopathy was identified as the most prevalent central neurological syndrome, followed by chemosensory disorders, while cases of stroke and encephalitis were less common. Our analysis showed that acute encephalopathy was associated with a higher rate of deaths, whereas having a chemosensory disorder was linked to survival.

Previous reports have associated smell and taste dysfunction with less severe disease and lower mortality.^9^^,^^22^ The frequency varied according to different reports, being higher among outpatients and when olfactory and gustatory tests measure smell and taste.^23^ In our study, whose participants are predominantly Black or mixed ethnicity, the frequency of olfactory and gustatory dysfunction was similar with other studies involving inpatients from different parts of Brazil.^24^^,^^25^ Compared with the rest of the sample, the group with chemosensory dysfunction was younger, predominantly female, with fewer comorbidities, characteristics associated with smell and taste dysfunction, and a better prognosis.^23^ This group had a lower frequency of serious illnesses. When they required ICU care, they needed less intensive support. Additionally, this group had lower levels of neutrophil-lymphocyte ratio, indicating less severe systemic inflammation, which aligned with other authors.^26^ A local immune response controls any viral invasion and replication.^27^ In COVID-19, this immune response, which damages the cells supporting olfactory neurons and results in smell loss^28^ may reduce the nasal load of SARS-CoV-2–2, which may affect RNAemia. RNAemia has been associated with dysregulation of the immune response and the severity of COVID-19.^29^^,^^30^

Participants with acute encephalopathy, compared to a group without acute encephalopathy, were characterized to be older, have severe disease, and have comorbidities, which align with previous reports.^8^^,^^10^^,^^31^, ^32^, ^33^ Unlike those with chemosensory dysfunction, they had a higher neutrophil-lymphocyte ratio, indicating systemic inflammation. The mechanisms that explain acute encephalopathy in COVID-19 are related to disease severity: systemic inflammation with cytokine storm, hypercoagulable state, hypoxemia, and other toxic or metabolic dysfunctions, such as electrolyte disturbances and multiple organ failure. However, direct nervous system virus invasion is uncommon.^34^^,^^35^ Another important characteristic of this group is that almost all participants were admitted to ICU. Furthermore, they required more intensive support. which certainly influenced a higher death occurrence in this group. Previous reports showed an association between mortality and acute encephalopathy.^21^ In Brazil, reports from different regions showed the same association.^24^^,^^25^ In addition, other authors associated acute encephalopathy of any cause with a higher risk of inpatient death during ICU stay, independently of etiology.^36^ We keep in mind that the rapidly growing number of cases has stressed health services during the pandemic. Many ICU units had to be set up quickly, and there was a shortage of qualified professionals, which may have contributed to many deaths.^37^

In a meta-analysis, Dorjee et al.^38^ reported 20 % (18 %‒23 %) of in-hospital deaths in the USA, 23 % (19 %‒27 %) in Europe, and 11 % (7 %‒16 %) in China. In China, a lower frequency of death, despite a more severe illness, was attributed to having fewer comorbidities and being younger than those in the US and Europe.^38^ In our sample, 35.4 % of participants died, the majority within 30 days. This frequency is higher than Dorjee et al.^38^ reported, but is in the range of deaths reported in hospitalized patients in Brazil.^13^ In Brazil, mortality was related to several aspects, such as socioeconomic inequalities, lack of access to health services, educational level,^39^ variability in the disease burden across different regions, and ethnicity.^13^ There is evidence that Black, Asian, and Minority Ethnic individuals had a higher risk of infection with SARS-CoV-2 and worse clinical outcomes, including ICU admission and mortality.^40^ Besides social vulnerability, genetic aspects may be involved in Black and *Pardos* susceptibility to developing severe disease.^41^^,^^42^ This study was conducted in a region with the highest Black population in our country (22.4 % Black, 57.3 % *Pardos*, 19.8 % White), according to the 2022 demographic census.^15^ These results do not demonstrate that ethnicity and educational level significantly influence neurological manifestation or mortality. However, this fact may be underestimated due to a lower frequency of White ethnicity in this sample. Conversely, a higher prevalence ratio of death was associated with severe disease, a higher neutrophil-lymphocyte ratio, cardiopathy, diabetes, and acute encephalopathy, those factors known as being associated with a worse prognosis. Some authors consider neutrophil-lymphocyte ratio as an independent risk factor of the in-hospital mortality for COVID-19.^43^

This study has limitations and potential biases inherent to an observational retrospective study. To begin with, since the findings relied on chart reviews, there may have been underreporting of symptoms in medical records for the following reasons: smell and taste dysfunction may not have been widely recognized, and many patients were seriously ill upon admission. In addition, there may have been misclassification of acute encephalopathy since most patients could not receive brain imaging or lumbar puncture. This can lead to underestimated stroke and encephalitis frequency, mainly if we consider that some patients were intubated and under sedatives. Finally, this is a unicentric study conducted during a limited timeframe, so the findings cannot be generalized to all patients admitted to the hospital, as they only represent a specific period throughout the pandemic.

The relevance of this study is underscored by several key factors. Firstly, these results corroborate previous findings from other studies that evaluated groups of hospitalized individuals. Secondly, the study highlights the neutrophil-lymphocyte ratio as an inexpensive and easy-to-obtain marker of inflammation severity and prognosis in a limited resource setting. Lastly, this study provides data on the neurological repercussions of the disease within a specific demographic context in a region with the largest Black population in Brazil. Our findings were similar to other studies done in different regions of Brazil. This suggests that the ethnic characteristics of this population do not significantly alter the neurological repercussions and their impact on the outcome of the disease.

In conclusion, this study found that COVID-19 is associated with neurological manifestations linked to different patient outcomes. Notably, individuals with chemosensory disorders experienced lower mortality rates, while those with acute encephalopathy faced higher mortality rates, regardless of race or educational level. Given Brazil's vast geographical diversity, various ethnicities, and different socio-economic backgrounds, it is relevant to understand how these aspects influence disease manifestation and prognosis in this context.

## Funding

This research did not receive any specific grant from funding agencies in the public, commercial, or not-for-profit sectors.

## Conflicts of interest

The authors declare no conflicts of interest.
